# Supplementary material for: Deletion of wfs1 Impairs Oligodendrocyte Precursor Cells Dorsal Distribution and Myelination Through the wfs1-hmgcs1 Axis in Zebrafish
Source: Biology (Basel). 2026 Aug 16;15(16):1402. doi: 10.3390/biology15161402 (PMC13510151; doi:10.3390/biology15161402)
Supplement: Supplementary file 1 [file biology-15-01402-s001.zip › File S1-Transcriptome sequencing data and differentially expressed genes (DEGs) in wfs1-- zebrafish at 5 dpf/1.GO/wfs1KOvsWT/GSEA/WT/TISSUE_HOMEOSTASIS/TISSUE_HOMEOSTASIS(GO_0001894).html]

Details for gene set TISSUE\_HOMEOSTASIS(GO:0001894)[GSEA]


|  || Dataset | deg.sample |
| Phenotype | sample.cls |
| Upregulated in class | WT |
| GeneSet | TISSUE\_HOMEOSTASIS(GO:0001894) |
| Enrichment Score (ES) | -0.46035814 |
| Normalized Enrichment Score (NES) | -1.8435103 |
| Nominal p-value | 0.0 |
| FDR q-value | 0.24539122 |
| FWER p-Value | 0.564 |
Table: GSEA Results Summary

  

Fig 1: Enrichment plot: TISSUE\_HOMEOSTASIS(GO:0001894)
  
Profile of the Running ES Score & Positions of GeneSet Members on the Rank Ordered List

  

| PROBE | DESCRIPTION   (from dataset) | GENE SYMBOL | GENE\_TITLE | RANK IN GENE LIST | RANK METRIC SCORE | RUNNING ES | CORE ENRICHMENT || 1 | 793830 | spata7 |  |  | 1223 | 1.875 | 0.0157 | No |
| 2 | 570321 | si:dkeyp-117h8.2 |  |  | 3297 | 0.974 | -0.0213 | No |
| 3 | 30741 | ctnna1 |  |  | 6114 | 0.575 | -0.0932 | No |
| 4 | 570583 | bbs12 |  |  | 6227 | 0.566 | -0.0804 | No |
| 5 | 559363 | syne2a |  |  | 6383 | 0.548 | -0.0694 | No |
| 6 | 325084 | src |  |  | 6875 | 0.502 | -0.0703 | No |
| 7 | 30269 | shha |  |  | 8146 | 0.415 | -0.0982 | No |
| 8 | 58122 | mcl1a |  |  | 8998 | 0.368 | -0.1144 | No |
| 9 | 406755 | rp2 |  |  | 9709 | 0.320 | -0.1274 | No |
| 10 | 64604 | nrf1 |  |  | 9962 | 0.306 | -0.1265 | No |
| 11 | 337298 | sulf1 |  |  | 10080 | 0.298 | -0.1216 | No |
| 12 | 560588 | cep290 |  |  | 10106 | 0.295 | -0.1139 | No |
| 13 | 394247 | pafah1b1b |  |  | 10333 | 0.283 | -0.1128 | No |
| 14 | 406281 | cluap1 |  |  | 11186 | 0.234 | -0.1328 | No |
| 15 | 323490 | ptbp1a |  |  | 11210 | 0.232 | -0.1268 | No |
| 16 | 405785 | tnfa |  |  | 11720 | 0.205 | -0.1369 | No |
| 17 | 100001171 | gpr137ba |  |  | 12154 | 0.182 | -0.1453 | No |
| 18 | 474330 | prrc1 |  |  | 12357 | 0.171 | -0.1467 | No |
| 19 | 564004 | usp45 |  |  | 12985 | 0.141 | -0.1623 | No |
| 20 | 559010 | exoc5 |  |  | 13041 | 0.138 | -0.1601 | No |
| 21 | 432389 | ift172 |  |  | 13274 | 0.127 | -0.1637 | No |
| 22 | 568195 | napbb |  |  | 13725 | 0.106 | -0.1748 | No |
| 23 | 114401 | bcl2l1 |  |  | 13894 | 0.097 | -0.1773 | No |
| 24 | 30701 | lef1 |  |  | 14523 | 0.068 | -0.1950 | No |
| 25 | 100000104 | stra6l |  |  | 14734 | 0.059 | -0.1999 | No |
| 26 | 402935 | chm |  |  | 14744 | 0.058 | -0.1985 | No |
| 27 | 393845 | pde6c |  |  | 15307 | 0.032 | -0.2153 | No |
| 28 | 406826 | emc3 |  |  | 16986 | -0.033 | -0.2670 | No |
| 29 | 567949 | piezo1 |  |  | 17044 | -0.034 | -0.2678 | No |
| 30 | 30510 | tcf7l2 |  |  | 17360 | -0.048 | -0.2764 | No |
| 31 | 492342 | mllt10 |  |  | 17734 | -0.064 | -0.2862 | No |
| 32 | 386932 | poc1b |  |  | 17847 | -0.069 | -0.2877 | No |
| 33 | 406350 | uhrf1 |  |  | 18151 | -0.083 | -0.2949 | No |
| 34 | 100124651 | siglec15l |  |  | 18808 | -0.114 | -0.3122 | No |
| 35 | 404620 | cdipt |  |  | 18977 | -0.120 | -0.3140 | No |
| 36 | 768133 | ezh2 |  |  | 19275 | -0.134 | -0.3194 | No |
| 37 | 556597 | bloc1s1 |  |  | 19709 | -0.153 | -0.3286 | No |
| 38 | 406689 | mcoln1a |  |  | 20236 | -0.176 | -0.3401 | No |
| 39 | 566470 | gdf6a |  |  | 20282 | -0.178 | -0.3363 | No |
| 40 | 336839 | cdc42 |  |  | 20515 | -0.189 | -0.3382 | No |
| 41 | 796551 | tert |  |  | 20891 | -0.209 | -0.3439 | No |
| 42 | 492496 | nr2e3 |  |  | 21502 | -0.241 | -0.3561 | No |
| 43 | 559945 | dot1l |  |  | 21571 | -0.244 | -0.3512 | No |
| 44 | 567911 | nxnl1 |  |  | 21687 | -0.251 | -0.3476 | No |
| 45 | 494087 | nxnl2 |  |  | 21798 | -0.257 | -0.3436 | No |
| 46 | 393910 | sulf2a |  |  | 22393 | -0.289 | -0.3539 | No |
| 47 | 795916 | mcoln1b |  |  | 23918 | -0.387 | -0.3906 | No |
| 48 | 415225 | dram2b |  |  | 24535 | -0.420 | -0.3979 | No |
| 49 | 394141 | dctn2 |  |  | 24688 | -0.429 | -0.3902 | No |
| 50 | 561701 | adora2aa |  |  | 25225 | -0.461 | -0.3938 | No |
| 51 | 414929 | ift57 |  |  | 25908 | -0.521 | -0.4002 | No |
| 52 | 503739 | hsf4 |  |  | 26039 | -0.533 | -0.3889 | No |
| 53 | 405826 | ift122 |  |  | 26861 | -0.614 | -0.3969 | No |
| 54 | 557044 | si:ch211-69e5.1 |  |  | 27378 | -0.657 | -0.3942 | No |
| 55 | 568520 | crb2b |  |  | 28973 | -0.912 | -0.4179 | No |
| 56 | 30590 | tp53 |  |  | 30325 | -1.401 | -0.4199 | Yes |
| 57 | 100006927 | si:dkey-11p23.7 |  |  | 30632 | -1.615 | -0.3829 | Yes |
| 58 | 436725 | acp5b |  |  | 30758 | -1.726 | -0.3370 | Yes |
| 59 | 567428 | vwa2 |  |  | 30899 | -1.863 | -0.2877 | Yes |
| 60 | 563428 | fras1 |  |  | 31617 | -3.325 | -0.2142 | Yes |
| 61 | 322056 | sulf2b |  |  | 31633 | -3.392 | -0.1168 | Yes |
| 62 | 100151323 | lca5 |  |  | 31773 | -4.324 | 0.0037 | Yes |
Table: GSEA details
[plain text format]

  

Fig 2: TISSUE\_HOMEOSTASIS(GO:0001894)
  
Blue-Pink O' Gram in the Space of the Analyzed GeneSet

  

Fig 3: TISSUE\_HOMEOSTASIS(GO:0001894): Random ES distribution
  
Gene set null distribution of ES for
 **TISSUE\_HOMEOSTASIS(GO:0001894)**
